# Left bundle branch pacing in patients with structural heart disease: personalizing cardiac resynchronization therapy

**DOI:** 10.1093/europace/euaf154

**Published:** 2025-07-29

**Authors:** Jacqueline Joza, Justin Luermans, Vartan Mardigyan, Haran Burri, Marek Jastrzębski, Pugazhendhi Vijayaraman, Kevin Vernooy

**Affiliations:** Department of Medicine, McGill University Health Centre, 1001 Decarie Boulevard, Montréal, Québec, Canada, H4A 3J1; Department of Cardiology, Cardiovascular Research Institute Maastricht (CARIM), Maastricht University Medical Center, Maastricht, The Netherlands; Department of Medicine, Jewish General Hospital, Montreal, Canada; Cardiac Pacing Unit, Cardiology Department, University Hospital of Geneva, Geneva, Switzerland; First Department of Cardiology, Interventional Electrocardiology and Hypertension, Jagiellonian University, Medical College, Krakow, Poland; Geisinger Heart Institute, Geisinger School of Health Sciences, Wilkes-Barre, PA, USA; Department of Cardiology, Cardiovascular Research Institute Maastricht (CARIM), Maastricht University Medical Center, Maastricht, The Netherlands

**Keywords:** Cardiac resynchronization therapy, Conduction system pacing, Left bundle branch pacing, Heart failure, Biventricular pacing, Left bundle branch block, Intraventricular conduction delay

## Abstract

Biventricular pacing remains the cornerstone of cardiac resynchronization therapy (CRT) in patients with heart failure, with well-established benefits. Left bundle branch pacing (LBBP) offers a physiologic alternative by engaging the native conduction system to restore synchrony and has generated significant enthusiasm. However, the growing adoption of LBBP should be tempered by recognition that a one-size-fits-all approach may not address the underlying substrate, particularly in those with intraventricular conduction delay. While a less-than-optimal LBBP implant may be sufficient in bradycardia patients, its adequacy in heart failure patients, who may require more precise consideration of conduction disease, remains uncertain. This review gives a comprehensive framework for integrating LBBP into CRT, including pre-implant, intraprocedural, and post-implant assessment. It also provides practical guidance on when to pursue LBBP alone, when to supplement with a coronary sinus lead, and when to consider conventional biventricular pacing, with an emphasis on a personalized approach to the underlying conduction substrate for maximal therapeutic benefit.

## Graphical Abstract

**Graphical Abstract:**
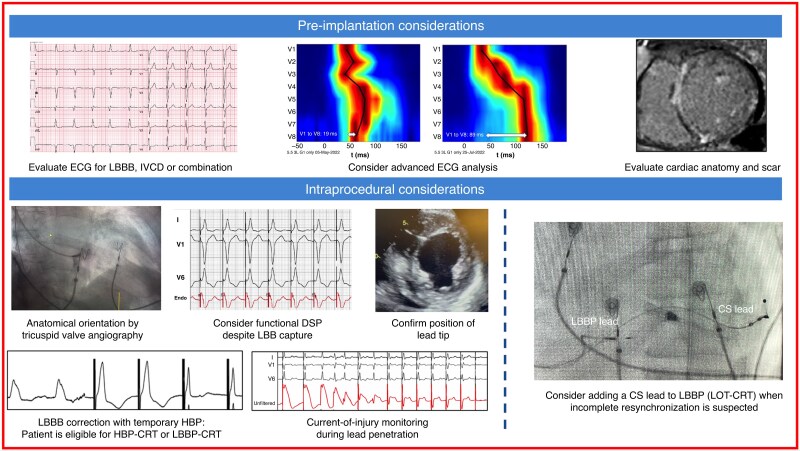

What’s new?

- This review emphasizes tailoring of cardiac resynchronization therapy based on the patient’s underlying conduction system substrate, including advanced pre- and intraprocedural decision-making regarding the choice of therapy (conduction system pacing vs. biventricular pacing vs. HOT or LOT-cardiac resynchronization therapy) to provide optimal clinical benefit to the patient.
- A comprehensive set of criteria to confirm left bundle branch capture criteria, particularly in heart failure patients with diseased conduction systems will be discussed.
- The concept of functional vs. anatomic deep septal pacing is introduced.
- Novel intraprocedural methods of performing left bundle branch pacing in heart failure patients as well as optimal programming post-implant are highlighted.

## Introduction

**Figure 1:**
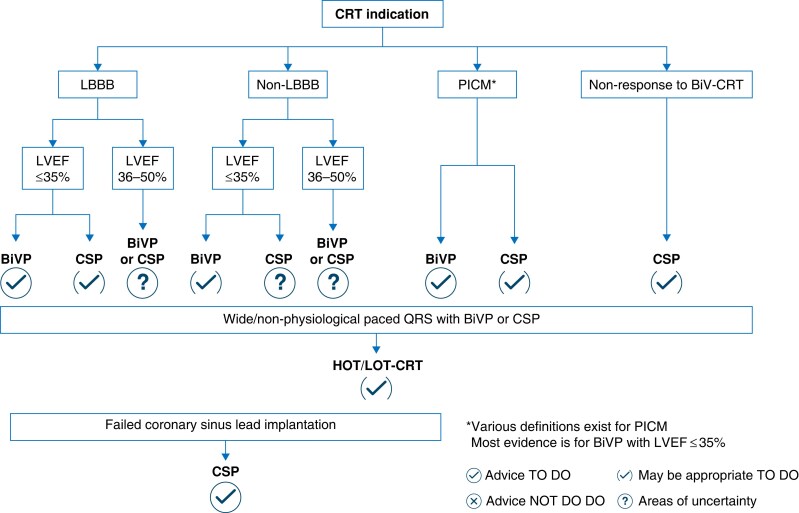
The 2025 ESC/EHRA consensus guidelines on conduction system pacing favour BiVP over CSP for traditional CRT indications.^1^

Until very recently, cardiac resynchronization therapy (CRT) has been primarily delivered through biventricular pacing (BiVP) and remains first line for patients with a CRT indication (*Figure 1*).^1^ Growing evidence suggests that a more targeted strategy addressing the underlying conduction abnormality offers a superior outcome. The interest in conduction system pacing (CSP) for CRT is fuelled by its more physiological approach, now recognized as an alternative form of CRT traditionally synonymous only with BiVP. After initial experience with His bundle pacing (HBP), left bundle branch pacing (LBBP) has now revolutionized the field of cardiac pacing.^2^ While LBBP implantation may be more straightforward in structurally normal hearts, the success rate and its potential to achieve complete resynchronization in CRT populations are lower and have likely been overestimated,^3–5^ underscoring the additional challenges presented by this complex patient cohort. This review aims to highlight important considerations in LBBP, while exploring strategies to enhance the success of conduction system capture while addressing the nuances and limitations of this evolving approach in patients with a CRT indication.

## Pre-procedural considerations

While the EHRA physician survey confirmed that BiVP remains the preferred strategy for patients with a CRT indication,^6^ an increasing number of physicians are adopting LBBP as a primary implantation strategy. This shift raises important questions about the need for a more tailored approach to patient selection and pacing strategy, emphasizing the importance of individualized decision-making. It becomes increasingly apparent that careful pre-procedural planning is essential when selecting the most appropriate CRT strategy; deciding in advance whether an additional coronary sinus (CS) lead is needed prevents a trial-and-error approach, which often leads to suboptimal outcomes. Electrocardiogram (ECG) findings, baseline imaging studies, and other relevant pre-implantation assessments play a crucial role in guiding the decision-making process to determine whether an initial BiVP-CRT vs. CSP-CRT approach or a combination is most appropriate (*Table 1*).

**Table 1:** Considerations for LBBP-CRT implantation

Pre-implantation considerations			
LBBB/IVCD	ECG	Evaluate Strauss criteria	Consider CS lead implantation (LOT-CRT) in the presence of IVCD
	VCG	QRS area > 95	
	UHF-ECG	Interventricular dyssynchrony, V1–V6 time	
Anatomy	Echocardiography	Dilatation of right heart chambers	Consider sheath with longer curve and more support
	CMR	Septal scar	Less suitable for LBBP, consider BVP-CRT
		LV lateral scar	Less suitable for CS pacing, consider CSP-CRT

Intraprocedural considerations			
Access	Venous access	Axillary/subclavian puncture may at times be preferred	A more proximal access provides more length for sheath manoeuvring
			Avoids sheath kinking with cephalic vein access
Site of conduction block	Right-sided EP study	His bundle mapping: LBBB correction	In case of IVCD, be prepared for CS lead or BiVP-CRT
	Left-sided EP study	Intra-LV left His–Purkinje mapping to confirm LBBB and correct LBBB by LBBP	In case of IVCD, prepare for CS lead or BiVP-CRT
Lead deployment	Locate His to determine LBBP target area	His bundle mapping	Use HBP to evaluate LBBB correction
		TV angiogram in RAO: His in summit of TV	Avoid screwing lead in TV by retracting guiding to LBBP target area
	Fluoroscopic approach	Around the cross of Zones 23, 5, and 6 of the nine-partition method	Estimation of TV annulus necessary
	Position on septum	Sheath support 1–2 cm distal from His bundle	Longer and stiffer sheath with dilated chambers
		Paced QRS morphology with intermediate axis and QS in V1	Aim for basal (to mid) septal position
	Lead advancement	ECG: continuous pacing with QRS morphology change	QRS morphology changing from QS to Q/R′ in V1
			Morphology of premature ventricular beats revealing lead depth
		EGM (unfiltered) for current of injury	Lead advancement till current of injury decreases significantly; reposition in case of perforation
		Fluoroscopy	Evaluate lead advancement in LAO
	Lead stability	Feedback from lead rotations	If no advancement with screwing: Entanglement = no torque transmission: clean screw and reposition lead Drill effect = rotations without advancement: reposition lead
Confirm LBB capture	Continuous pacing	ECG: changing QRS morphology	Further change in QRS with LBB capture
		ECG: changing T-wave	Normalization of repolarization with LBB capture
	Threshold test	Change in V6 RWPT and V1 RWPT with decreasing output	nsLBBP->sLBBP: V1 RWPT delays at similar V6 RWPT
			nsLBBP->LVSP: V6 RWPT delays
	Individualized V6 RWPT	Comparing paced with native QRS	LB potential to V6 RWPT is similar to stimulus to V6 RWPT
			Native V6 RWPT = paced V6 RWPT
	QRS morphology	V6 RWPT	<74 in normal heart, <80 in wide QRS Can be underestimated by LV fascicular pacing
		V6 to V1 time	>44 ms LBBP

Post-procedural considerations			
Pacing device	Individualized device based on leads and clinical characteristics	Dependent on PM/ICD, DF4, or DF1 shock lead, presence of atrial lead and additional CS lead	More cost-effective to use simpler devices, but less heart failure diagnostics and capping of leads results in MRI non-compatibility
Device programming	LBBP programming	Unipolar vs. bipolar LBBP	Anodal capture in LBBP associated with less improvement
	Fusion pacing	AV optimization in patients with intact AV conduction	Merge activation from LBBP with activation via RBB conduction

## Assessment of the electrical substrate

The feasibility of CSP for CRT is supported by the observation that the site of block in patients with left bundle branch block (LBBB) is often located within the His bundle or at the very proximal LB, making it therefore potentially amenable to both HBP and LBBP.^7^ However, intraventricular conduction delay (IVCD) representing predominantly intramyocardial delay—rather than true conduction block—can complicate ECG interpretation and obscure the distinction from true LBBB.^8^ The limited sensitivity and specificity of existing LBBB definitions with disagreement between LBBB definitions result in frequent misclassification, with disagreement even among experts.^8,9^

The complexity is illustrated by a study in which in a cohort of 72 patients meeting the criteria for LBBB, 36% demonstrated intact activation over the left bundle branch on left-sided electrophysiology (EP) study. This means that 36% had pure IVCD masquerading as LBBB, potentially making these patients less likely for successful resynchronization using CSP. When the block was localized to the left His bundle (72%) or a more proximal LBB (28%), HBP corrected the wide QRS with recruitment of the Purkinje fibres in only 94% and 62% of cases, respectively.^7^ This does not imply that all IVCDs are not amendable to correction with CSP. Intraventricular conduction delays due to proximal myocardial delay may be correctable with CSP (i.e. heterogeneous substrate including both bundle branch block with altered myocardial substrate, for instance, in the case of myocardial infarction). Blocked fascicles often coexist with IVCD where distal LBBP may correct it. Even if the IVCD is not corrected, preserving and controlling the timing of LV activation with CSP opens possibilities with LOT-CRT. A one-size-fits-all approach to resynchronization, relying exclusively on HBP or LBBP, may be less optimal. Without a deeper understanding of the electrical substrate, we risk misdiagnosing and mismanaging patients, ultimately compromising the efficacy of resynchronization therapies.

The evaluation of intracardiac QRS correction with HBP has also provided insights into the diagnostic accuracy of various ECG criteria for LBBB. A 100% sensitivity and 42% specificity were found for mid-QRS notching, 79% sensitivity and 45% specificity for QRS width > 150 ms, and 62% sensitivity and 39% specificity for the absence of an R wave in V1.^7^ Although the ECG criteria for LBBB remain debated (see Supplementary material online, *Table S1*), the Strauss criteria seem to provide the highest overall diagnostic reliability.^7^ Electrocardiogram characteristics to identify LBBB beyond Strauss criteria are noted in Supplementary material online, *Table S2*.^10^

The Strauss criteria are still far from perfect. An analysis of combined cohorts of both post-transcatheter aortic valve replacement (TAVR) LBBB and corrected LBBB during CSP (defined as narrowing of the QRS complex on all 12 leads to either <120 ms or a QRS width reduction of at least 20%)^11^ demonstrated that 10% of patients would not have met Strauss’s QRS duration criteria (>130–140 ms).^12^ Conversely, the upper limit for a QRS has not been defined, but considering that a 52 ms increase in QRS duration was noted with TAVR-induced LBBB, a QRS duration > 170 ms likely harbours additional underlying IVCD. Assessment of serial ECGs is essential, paying close attention to notching and slurring, which are indicative of electrical dyssynchrony. While true LBBB can typically be corrected with CSP, cases of pure IVCD or a combination of IVCD and LBBB will necessitate the use of a CS lead in most instances, either as a primary strategy or in conjunction with CSP.

**Figure 2:**
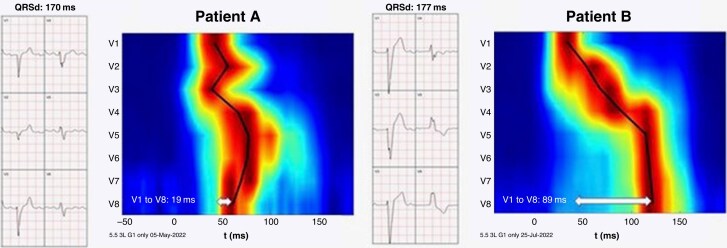
Ultra-high-frequency ECG demonstrating activation patterns for (*A*) IVCD and (*B*) true LBBB.^15^

Over the past decade, alternative methods measuring ventricular dyssynchrony have been studied. These methods range from relatively simple options such as vectorcardiography and ultra-high-frequency ECG (UHF-ECG) to more advanced techniques like the ECG belt, ECG imaging, and electro-anatomic mapping.^13^ Epicardial propagation mapping with ECG imaging has been shown to identify those LBBB which are amenable to HBP.^14^ The UHF-ECG technique is unique due to its ability to rapidly and non-invasively perform dyssynchrony analyses, making it potentially very attractive for implementation in daily practice.^13,15^  *Figure 2* shows two examples of UHF-ECG from patients with a wide QRS complex. Patient B clearly shows a delayed LV lateral activation consistent with LBBB, suggesting response to CSP or to BiVP. However, whether UHF-ECG can discriminate between LBBB and IVCD or even those with a combination of LBBB and IVCD needs to be studied.

## Pre-implant imaging

**Figure 3:**
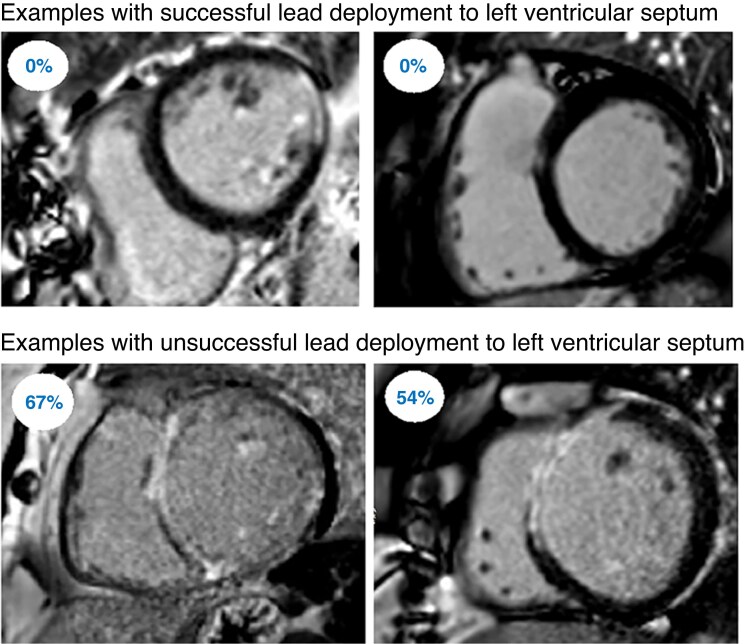
Septal scar represented by percentage of LGE preventing successful lead deployment at the left ventricular septum.^20^

Imaging-guided lead placement is increasingly recognized as important not only for BiV-CRT but also for CSP to identify scar location to maximize the potential for improvement.^16^ Larger LV end-diastolic dimension and septal late gadolinium enhancement (LGE) independently predict non-response to LBBP-CRT, even in patients meeting Strauss criteria for LBBB.^17–19^ In 25 cardiomyopathy patients undergoing LBBP with demonstrated LGE on cardiac magnetic resonance (CMR), the fixed helix lead could not penetrate the septum to reach the LV subendocardium in nine patients.^18^ Transmural scar in the so-called LBBP zone defined as the overlapping areas of the inferior aspect of the antero-septum and the superior aspect of the infero-septum predicted procedural failure (*Figure 3*). Procedural failure was not statistically significant in patients with ischaemic vs. non-ischaemic cardiomyopathy. In BiV-CRT, LGE-CMR can also predict non-response by identifying a scar near the LV lead deployment site in the postero-lateral wall, which prolongs paced QRS duration and hinders resynchronization,^21^ where positioning the lead away from the scarred myocardium is associated with improved outcomes.^22^ Recent prospective observations from the MADURAI-LBBP study demonstrated that scar burden (≤10%) determined by CMR can predict CRT response to LBBP and the need for defibrillator therapy.^23^

## Other pre-implant considerations

In selected patients, LOT-CRT may offer meaningful benefit, despite the need for an additional transvenous lead. Deferring the decision to implant an additional lead to a time after follow-up with a ‘wait-and-see’ strategy often leads to suboptimal outcomes and exposes patients to higher risks of future upgrade procedures including infection, complications, and encountering vein occlusion requiring extraction prior to upgrade. A pragmatic, low-risk approach is where CSP is primarily attempted, with the option to add a CS lead should resynchronization be insufficient. Otherwise HOT- or LOT-CRT may be considered for all CRT-pacemaker patients until evidence demonstrates the benefit of a stand-alone CSP approach in this population. Shared decision-making is also essential, as the goals of a 50-year-old with a left ventricular ejection fraction (LVEF) of 35% differs markedly from those of a patient with an LVEF of 5%, where short-term prognosis and lead burden may outweigh long-term considerations of extra transvenous leads. Recent reports of the feasibility of placing traditional defibrillator leads or novel, small-diameter leads in the LBBP location increase the opportunity to provide LOT-CRT with a three-lead approach but require careful planning.^24,25^

## Intraprocedural decision-making

### Intraprocedural determination of the location of block

**Figure 4:**
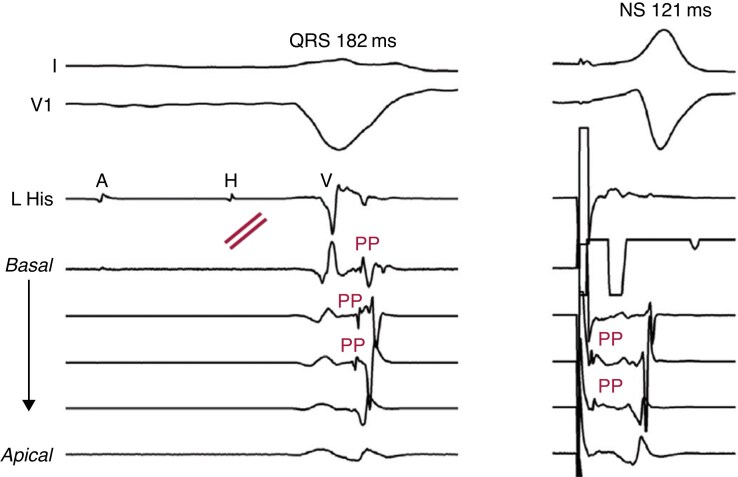
Left: Left-sided EP study with demonstration of complete conduction block at the proximal left bundle with QRS activation over the right bundle with retrograde activation of the Purkinje potentials (PPs) from the apex to the base of the left ventricular septum. Right: QRS correction with recruitment of latent PP during non-selective HBP with base to apical activation. Correction of the LBBB during HBP identifies patients who are likely to achieve maximal electrical resynchronization with LBBP.^26^

Determining the level of conduction block is essential in determining the best CRT strategy. The level of block in suspected LBBB can be best defined intraprocedurally. By employing a left-sided EP study with a duodecapolar catheter retrogradely placed in the LV at the interventricular septum, one can identify the ventricular activation pattern and site of conduction block.^26^ Where normal ventricular activation is from apex to base, with Purkinje potentials preceding the ventricular electrogram (EGM), the presence of a left-sided split His (focal left intrahisian conduction block) or block within the left bundle with disrupted Purkinje activation indicates the presence of a true LBBB. With stimulation distal to the site of block, correction of the LBBB is observed, with QRS narrowing and pre-systolic recruitment of the latent Purkinje potentials immediately after the pacing stimulus (*Figure 4*). In contrast, the presence of delayed but intact Purkinje activation from apex to base is suggestive of IVCD. Although invasive and not practical in routine clinical practice, the study *may* be warranted when considering the permanency of lead placement and the critical importance of delivering optimal CRT.

**Figure 5:**
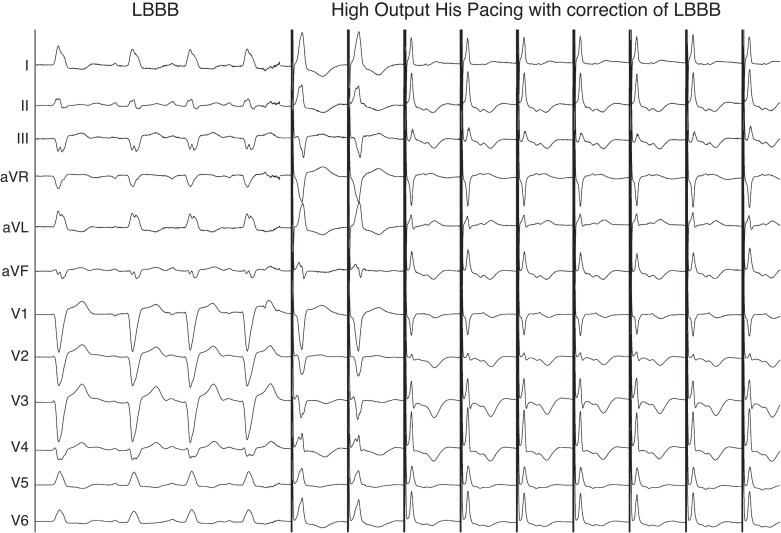
Intraprocedural high-output pacing at the level of the His corrects the underlying LBBB.

The second method to define the level of block is to demonstrate LBBB correction by temporary right-sided HBP prior to LBBP lead implantation (*Figure 5*).^27^ This may be achieved by direct His pacing with an EP catheter or with the LBBP lead during implantation. If complete correction is achieved, then CSP should be considered an appropriate pacing strategy. His bundle pacing will also provide the lateral activation time [V6 R-wave peak time (RWPT)] target value to achieve conduction system capture during LBB pacing: the stimulus to V6 RWPT during LBBP should be at least ∼10 ms shorter than the stimulus to V6 RWPT during HBP (81% sensitivity and 100% specificity).^27^

Finally, although not advised to aim for, incidental mechanical bumping of the right bundle that induces complete heart block, in general, suggests the presence of LBBB with rare exceptions.^28^ Conversely, if heart block does not occur and a wider, atypical right bundle branch block becomes apparent, then this may be more indicative of the presence of at least some underlying IVCD, although again not universally the case.

### Assessing electrical response to conduction system pacing

**Figure 6:**
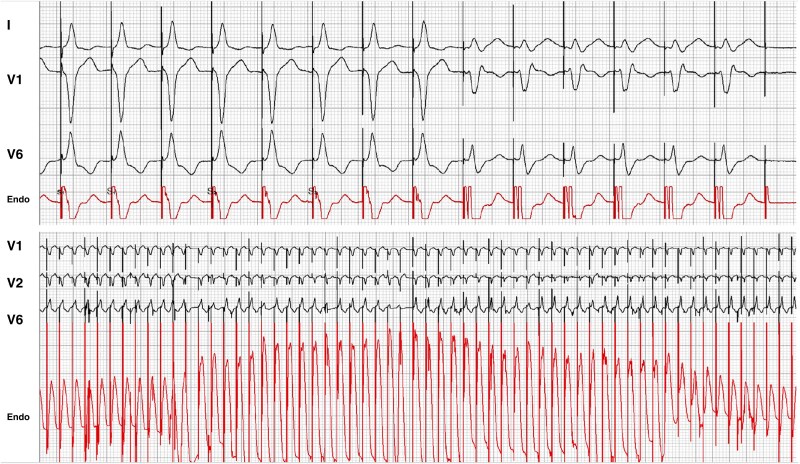
Demonstration of non-selective to selective LBB pacing (transition) in an ischaemic cardiomyopathy patient with a very dilated left ventricle despite the QRS morphology in V1 of only DSP (during NS-LBBP), a phenomenon referred to as ‘functional’ DSP due to slow conduction via the left conduction system.^42^ In such a case, a CS lead should be considered because of incomplete resynchronization. (Permissions obtained to reproduce figure.)

**Figure 7:**
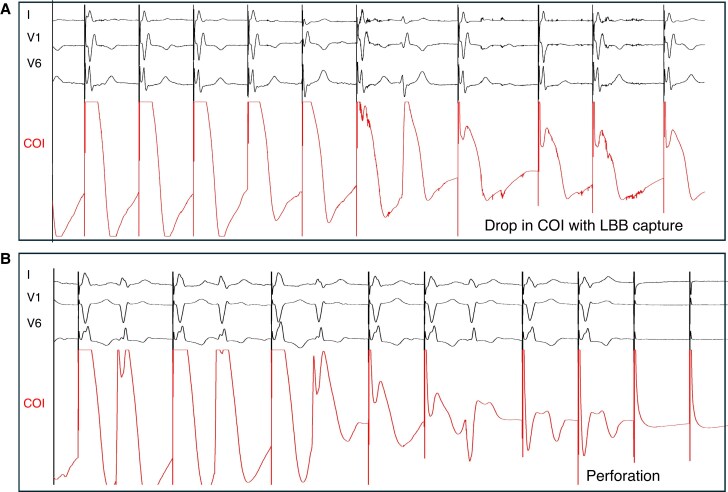
(*A*) Achievement of LBB pacing with reduction in COI coinciding with LBB capture. Although the presence of a terminal R/r in V1 at the left of the screen may have suggested to stop, the COI remained high, and therefore screwing was continued until a reduction in COI was seen. (*B*) Reduction in COI during screw progression through the septum. Only functional DSP obtained despite progression through the entire septum through to perforation to the left ventricle (as exhibited by loss of capture on the final beat).

True LBB capture has been shown to be present in only about 60% of CRT implantations.^29^ A retrospective observational study initially suggested equivalency between left ventricular septal pacing (LVSP) and LBBP in terms of LVEF improvement, rates of HF hospitalizations, and overall survival at a median of 10 months.^30^ Conversely, larger prospective studies with longer follow-up have suggested superiority of LBBP as compared to both biventricular pacing (BVP) and LVSP, though these findings are limited by the lack of randomization and higher-than-expected rates of CS lead failure.^31,32^ Left bundle branch pacing capture is essential, and thus, a deep electrophysiological understanding of the criteria is necessary to differentiate LVSP from LBBP (see *Table 2* for a comprehensive approach). As the pre-systolic LBB potential is absent (except for rare cases)^40^ in patients with LBBB, preventing the use of physiologic-based criteria (LBB potential-to-peak V6 interval), achieving a transition pattern should be prioritized. Transition is typically observed near the capture threshold during unipolar threshold testing. Notably, this transition may still occur even when the ECG only resembles deep septal pacing (DSP) (*Figure 6* as well as discussion below). Some transitions may be challenging to interpret as they may represent either bona fide transition to selective capture of a diseased part of the LBB conduction system with substantial latency; or alternatively, they may represent myocardial-to-myocardial capture transition where strands of the myocardium are separated by fibrous tissues, although quite rare.^41,42^ In the absence of a clear transition pattern, the V6 RWPT is helpful. Although no definitive benchmarks exist for lateral wall activation in HF patients, it has been proposed that achieving a V6 RWPT < 80–82 ms with proximal LBB lead positioning likely indicates LBB capture although with low sensitivity, based on clinical experience and data from observational studies^43^ and small randomized trials.^44^ Although proximal LBB capture should be the goal, insufficient sheath support in larger hearts may direct the lead to the fascicular region. This can result in paced QRS complexes with an R/S morphology in V6 and short V6 RWPT with prolonged V6–V1 peak times, creating a misleading impression of LBB capture.^45^ During fascicular pacing, a line may be drawn on the 12-lead recording system from the peak of V6 up to the peak of aVL and lead I. This may clearly demonstrate a deceptively short V6 RWPT. In this instance, the global RWPT should be considered where the combination of RWPT in lead I + V6 should be <162 ms (sensitivity 96% and specificity 94%) for a healthy conduction system and <187 ms (sensitivity 88% and specificity 80%) for a diseased left conduction system.^37^ Finally, continuous pacing during lead penetration with a displayed current of injury (COI) has proven to be very useful to predict impending perforation and can be added to the EP recording system with an unfiltered EGM for COI monitoring during lead deployment (typically 0.5–500 Hz).^46^ Pacing at a higher rate with shorter sweep speeds during screwing will permit visualization of an initial COI increase with DSP, development of r′ in V1 within the LV septum, and finally a reduction in COI when the LV endocardial septum is reached where LBB capture can be obtained (*Figure 7A*). In some cases, however, despite reaching the LV endocardium, pacing does not necessarily result in LBB capture (*Figure 7B*) and not infrequently only a DSP morphology or LVSP with tiny terminal V1 R morphology can be obtained prior to perforation.

**Table 2:** Overview of electrophysiological criteria for confirming left bundle branch capture

1. QRS transition (gold standard)^33^
Unipolar threshold testing either with (a) non-selective to selective (NS to S) or (b) non-selective to LV septal transition (NS to LV septal). Transition typically occurs at near threshold. a) NS → S: no change in V6 RWPT (or shortening), increase in R′ amplitude/width in V1, development or increase in amplitude of S, and/or decrease in amplitude of R in leads I, aVL, and V5, V6, and discrete signal in the LBB-filtered electrogram b) NS → LV septal: increase in V6 RWPT by ≥10–15 ms, reduction in the R′ amplitude/width in V1, disappearance/reduction of S, and/or increase in amplitude of R in leads I, aVL, and V5 and V6
2. QRS transition during programmed electrical stimulation
Transition may also be observed with unipolar programmed stimulation^34^ that exploits the refractory periods of the myocardium and LBB. Both myocardial and LBB-selective responses can be obtained; however, selective response is by far easier to interpret and 100% diagnostic being equivalent to QRS transition during threshold test. Selective LBBP response: slow drive train followed by rapid double extra-stimuli (e.g. S_1_1000–S_2_400–S_3_400 ms) will prolong the myocardial refractoriness resulting in a beat typical of selective LBBP capture. Selective response is most easily obtained with delivering double extra-stimuli on intrinsic rhythm (which in a way is the slowest possible ‘drive’), for example, S_1_400 + S_2_400 ms, with S_2_ progressively shortened until refractoriness is reached or selective response obtained. If this does not result in selective response, sometimes a small change of the coupling interval length will result in obtaining selective response (e.g. S_1_380 + S_2_ 380, or S_1_420 S_2_ 420 ms).
3. Physiology-based criteria
LBB potential to V6 RWPT = distance from the pacing artefact to V6 RWPT (+10 ms).^35^ If the LBB potential is not visible, QRS onset may be used for measurement both during native rhythm and paced rhythm.^55^ In cases where there is no native narrow QRS that can serve as a reference, this often times can be obtained with His-corrective pacing with high-output amplitude. In such cases, the V6 RWPT should be ∼10 ms shorter with LBBP than with the V6 RWPT during His-corrective pacing.
4. V6 RWPT: shortening of V6 RWPT
Shortening by ≥10 ms during deep septal pacing with R′ in V1 followed by short and constant V6 RWPT at low and high unipolar output when the lead is advanced deeper^27,36^ Target: a) <74 ms if intrinsic QRS narrow or isolated RBBB b) <80 ms if intrinsic QRS wide (i.e. LBBB, IVCD, bifascicular block, wide escape, or asystole) (<100 ms less specific)
5. Global RWPT^37^
Useful for fascicular lead positions a) Combination of RWPT in I + V6: optimal cut-offs for healthy left conduction system: 162.5 ms (SN 96%, SP 94%) and 100% specific cut-off 148.5 ms (SN 69.1%). Optimal cut-offs for diseased left conduction system: 187.5 ms (SN 88%, SP 80%) and 100% specific cut-off 163.5 ms (SN 52) b) Lead I RWPT: a value < 72 ms provides a 100% specificity for conduction system capture, and <81 ms provides an optimal sensitivity and specificity.^37^
6. V6–V1 inter-peak interval
Target: ≥44 ms 100% specific for LBBP^38^ Caveats: absence of an R′ in V1 will prevent V6–V1 measurement, and apical or posterior fascicle area pacing without LBB capture may result in rS morphology and give a ‘falsely’ prolonged V6–V1 time; therefore, it should be used only with R or Rs morphology in V6.
7. Current of injury (COI) drop during continuous pacing^39^
A rapid reduction in COI amplitude during lead penetration. While this phenomenon is not diagnostic of LBB capture in itself, it is diagnostic of reaching the left subendocardial area and helps to make the diagnosis of functional DSP/LVSP (see text for explanation).
8. Transition zones during continuous pacing^39^
Sudden (beat-to-beat) drop in QRS amplitude in V3–V6 (>0.3 mV) in two consecutive leads Sudden development of terminal s-wave in I and V5/V6 Sudden increase in terminal R/r wave in V1 Sudden normalization of repolarization abnormality in I and V5/V6 (ST-segment shift from downsloping to horizontal/upsloping, or t-wave reversal from negative to positive/biphasic) Sudden V6 RWPT shortening by 10 ms (in the V1 R zone)
9. Fixation beats
The morphology of the fixation beats (M beats, i.e. beats of rsR′ type) may be suggestive of LBB capture. -In patients with LBBB morphology, fixation beats observed during lead implantation usually have RBBB-type morphology and may be the only chance to observe the LBB potential which in such situation is suggestive of LBB capture and can be used for application of the physiology-based criteria (see Point 3 above).

EGM, electrogram; SN, sensitivity; SP, specificity.

Even when the lead tip has reached the LV subendocardium, the paced QRS may be suboptimal, which should prompt the operator to consider an additional CS lead (LOT-CRT) or to abandon LBBP altogether for BiVP (*Table 3*). The CSPOT study supports this approach where 48 HF patients prospectively underwent implantation of both LBBP and CS leads with simultaneous invasive haemodynamic measurement.^43^ Despite expert implantation with precise lead placement at the left subendocardial septum, DSP was achieved in a surprisingly significant proportion (44%) of cases; i.e. no terminal R/r was noted in V1 despite clearly reaching the LV endocardium. This concept of ‘functional vs. anatomic’ DSP was introduced by Jastrzebski *et al*.^42^ Functional DSP refers to the absence of a terminal R/r in V1 due to the severe conduction system slowing preventing effective activation, whereas anatomical DSP reflects the lead’s failure to physically reach the conduction system.^42,43^ In CSPOT, functional DSP was associated with longer V6 RWPT (103 ms vs. 82 ms for LBB capture), worse haemodynamics, and less QRS shortening compared to BiVP.^43^ In patients with a baseline QRS duration > 171 ms (LBBB or IVCD) or in whom only DSP was achieved, the addition of a CS lead for LOT-CRT resulted in acute haemodynamic improvement. This suggests that a considerable proportion of CRT candidates undergoing LBBP benefit from implantation of an additional CS lead (LOT-CRT). At 6 months, LOT-CRT demonstrated significantly greater LVEF improvement compared to BiVP (16.1% vs. 6.1%; *P* < 0.01).^47^

**Table 3:** Considerations of when to implant an additional CS lead during LBBP-CRT (LOT-CRT)

Baseline ECG	QRS morphology suggestive of IVCD rather than LBBB
	QRS duration over 170 ms suggestive of IVCD beyond the presence of LBBB
Imaging	Presence of septal scar
LBB pacing	Inability to reach the LV subendocardium: anatomical DSP
	LBB capture without typical LBB-paced QRS morphology: functional DSP
	V6 RWPT during LBBP > 90 ms
	Inability to capture the conduction system; LV septal pacing
Mapping	Inability to correct the underlying LBBB with temporary His bundle pacing
	Intact His–Purkinje signals on LV septal endocardium suggestive of IVCD
	Persistently delayed LV lateral wall activation during coronary sinus mapping after LBBP implant
	Non-invasive mapping using ECG-imaging or UHF-ECG
Haemodynamics	Lack of appropriate increase in blood pressure with LBBP

### Anatomic location of the lead tip

Positioning the lead at the LV septal subendocardium is critical in achieving LBB capture.^48^ This was confirmed in a study of 30 patients undergoing LBBP lead implantation under transthoracic echocardiographic guidance, where LBB capture was successfully achieved in 29 patients (97%), compared to 21 out of 30 patients (70%) using a fluoroscopic approach.^49^ Successful lead implantation (confirmed by presence of transition) was characterized by the lead tip positioned at the LV subendocardium in the echo group. Those undergoing fluoroscopic implant did not achieve such high success rates likely due to concerns about the risk of perforation after already achieving an R′ pattern in V1 and a V6 RWPT of <80 ms. Echocardiographic guidance also facilitated perpendicular sheath positioning, thereby improving lead advancement. Monitoring lead depth may also be evaluated using the COI on the unfiltered EGM during pacing while the lead is being screwed into the septum. An initial increase in COI is observed with penetration into the LV septum, with a clear reduction in COI when the LV subendocardium is reached (*Figure 7A*). A COI amplitude below 4 mV may be associated with perforation and warrants careful evaluation of the COI amplitude as the COI drop is occurring.^39^ Further lead rotations should be abruptly stopped once the COI drop occurs.

A QS pattern on the unfiltered EGM signal correlates with partial perforation as confirmed by echocardiography.^50^ The key indicator to differentiate between partial and complete perforation is primarily based on the amplitude of the COI. *Figure 7B* shows a case of perforation where the COI further decreases after a QS pattern is observed leading to loss of capture. Partial perforation was not associated with adverse clinical outcomes such as lead dislodgement or stroke.^50^

### Procedural challenges in heart failure patients: anatomical, access, and sheath considerations

A learning curve for LBBP exists, which will affect procedural success in less experienced hands.^3^ Understanding anatomical potential pitfalls and the strategies to overcome them is essential.

### Access

**Figure 8:**
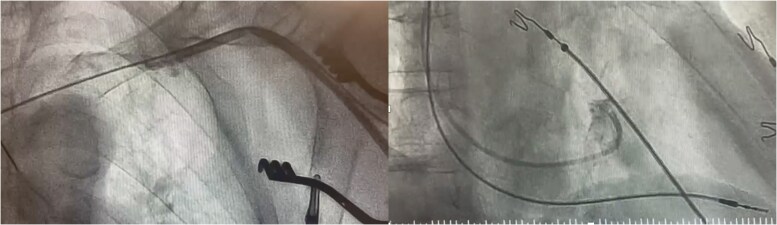
Kinking of the sheath can rarely occur during cephalic vein approach. Ultimately switched to axillary vein puncture for improved sheath reach and apposition.

While cephalic cut down is a common approach for venous access in pacing lead implantation, at times, it is suboptimal for LBBP. The required sheath length may be insufficient, particularly in tall patients or those with enlarged right-sided chambers, as the cephalic route necessitates additional length. Similarly, a subclavian or axillary venous puncture may offer more suitable access for LBBP in the setting of a tortuous cephalic vein. Regardless of the approach, kinking of the guiding sheath should be suspected when lead rotation and advancement become difficult (*Figure 8*). While kinking typically occurs proximally in the venous access sheath, it can also develop distally in the superior vena cava when substantial torque is applied. In such cases, switching to a stiffer delivery catheter or using a longer outer sheath may be necessary and CSP-CRT implanters should have the availability of a variety of CSP guiding catheters.

### Delineation of the target location

**Figure 9:**
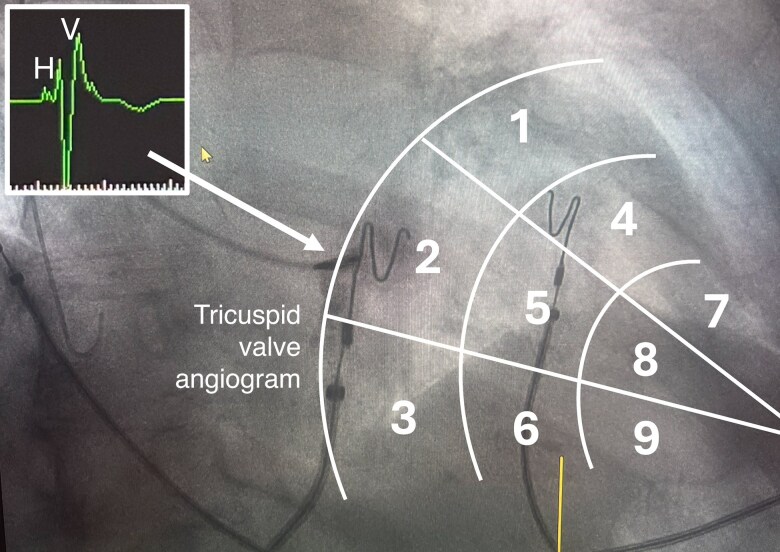
Tricuspid valve angiography with the nine-partition method for fluoroscopic-guided implant.

An angiogram of the tricuspid valve annulus (TVA) provides valuable insight when identifying the target area for LBBP. By injecting contrast directly at the TVA in a slightly right anterior oblique (RAO) projection, the angiogram will aid in locating (i) the tricuspid valve; (ii) the His bundle area, which is located at the summit of the tricuspid valve; and (iii) the target proximal LBBP area, which lies 1.5–2 cm distal of the His bundle. With larger contrast boluses, TVA angiography can also provide an assessment of right atrial (RA) and right ventricular (RV) dimensions (*Figure 9*). This technique helps operators to avoid placing the lead too close to the tricuspid valve, thereby preventing entrapment of the septal tricuspid leaflet. Determining the His bundle area can also be achieved without a contrast medium by mapping the His bundle itself, which should be especially considered in case of kidney dysfunction or allergy to contrast or when initially aiming for His corrective pacing.

The nine-partition method offers a purely fluoroscopic method for implant using fluoroscopic imaging without TVA angiography (*Figure 9*).^51^ This potentially faster approach proposes to eliminate the need for an EP recording system, though this is not the preferred method as the precise location of the TVA can differ significantly from expectations in cardiomyopathy patients.

### Sheath and lead manipulation

Failure of lead advancement deep into the septum may result from a suboptimal delivery sheath position on the interventricular septum. The delivery catheter should be positioned as perpendicular as possible on the septum and is a key factor for the successful implantation of a LBBP lead. One contributing factor to a suboptimal position is a significantly dilated RA and/or RV. The presence of RA/RV dilatation can be confirmed with a TVA angiogram as discussed above, which helps tailor the selection of a second sheath if the initial one proves insufficient for lead implantation (see Supplementary material online, *Video S1*).

**Figure 10:**
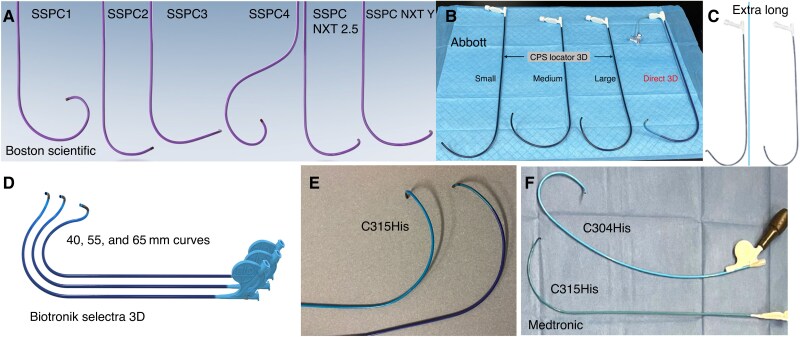
Available sheaths for conduction system pacing. (*A*) Boston Scientific sheaths: SSPC1–4: 40 cm working lengths, inner diameter 8Ff SSPC NXT 2.5 and Y working lengths 42 cm, inner diameter 7 Fr, and outer diameter 9 Fr. (*B*) Abbott sheaths: CPS locator 3D and direct 3D, working length 42 cm, inner diameter 7 Fr, and outer diameter 9 Fr. (*C*) Abbott CPS locator 3D medium and large extra-long, working length 45 cm, and inner and outer diameters 7 Fr and 9 Fr, respectively. (*D*) Biotronik Selectra 3D: two lengths (39 and 42 cm) and three curves (40, 55, and 65 mm). Inner diameter 7.3 Fr. (*E* and *F*) Medtronic C315His fixed sheath, 43 cm working length and inner and outer diameters 5.4 Fr and 7.0 Fr. C304His deflectable sheath working length 43 cm. Inner and outer diameters 5.7 Fr and 8.4 Fr.

Maintaining a variety of sheaths as part of the armamentarium (*Figure 10*) is essential to avoid being restricted to a single manufacturer or sheath type. An operator should begin with their preferred standard sheath; however, most delivery sheaths have a working length of 42–43 cm, which may be insufficient for certain anatomies. If the initial sheath does not provide adequate apposition, a careful next choice should be considered. In the presence of RA/RV dilatation, longer sheaths and larger curves may be required, with the latter typically advancing to the posterior mid-distal ventricle. Reshaping the guiding sheath can further optimize stability on the septum. A stiffer sheath might sometimes overcome instability of the sheath on the interventricular septum for adequate lead deployment. A final option currently offering the longest reach inside the RV cavity is the ‘sheath-in-sheath’ technique, using a CS guiding catheter as an outer sheath and the C315HIS as an inner sheath for enhanced stability in dilated hearts. However, since the C315HIS is too short to fully advance through a CS guide, it can be advanced through a small ‘nick’ created in the CS guiding catheter.

### Other considerations

If the lead remains difficult to advance into the septum, interference from the septal tricuspid leaflet should be suspected. To avoid trapping the septal leaflet, the delivery catheter can be advanced deeper into the right ventricle over a wire and then retracted under TVA angiography. Endocardial and myocardial fibrosis should be considered when lead advancement is challenging, as it may impede penetration. After several failed attempts, the lead tip should be inspected for tissues to be removed from the helix before making a next attempt. If advancement is unsuccessful, an alternative implantation site should be considered, accounting for varying lead behaviour. A promising approach for patients with a fibrous septum may be with the simple and short application (1 s) of high-power electrosurgery radiofrequency energy (55 W) to deliver the lead but confined to overcoming discrete fibrous barriers, with the remainder of lead deployment conducted in the conventional manner.^52^

**Figure 11:**
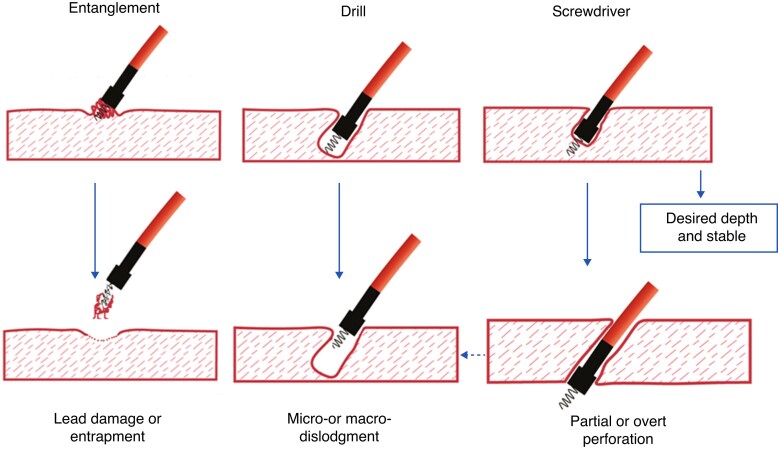
Lead behaviour during penetration of the interventricular septum. Both drill and screwdriver effects can result in perforation.^34^

Should there be strong torque build-up after the initial five rotations without torque transmission, the lead position should be changed as there is likely presence of entanglement (*Figure 11*).^53^ This can be confirmed after withdrawing the lead and observing excess tissues on the tip. If there is transmission of the driving force, then the response to pacing has to be monitored. With progressive paced QRS narrowing/morphology change in V1 (screwdriver effect), then rotations should be continued until LBB capture is obtained or the COI is declining. If there is no progression of the paced QRS and no lead movement to the LV endocardium despite transmission of the torque, then the drill effect should be suspected and the lead should be repositioned. An excessive number of rotations should always be avoided.

### Post-implantation considerations

Current cardiac implantable electronic devices are not specifically designed for CSP. Therefore, following the implantation of an LBBP lead as an alternative to BiVP-CRT or as a complementary resynchronization strategy (LOT-CRT), decisions must be made regarding the appropriate generator type and lead port connection. In the CRT population, these choices depend on factors such as underlying rhythm and intact atrioventricular (AV) conduction, the indication for an implantable cardioverter defibrillator (ICD), and the presence of an additional ventricular backup or CS lead (the latter for LOT-CRT). This results in multiple possible lead connections.^54^ Each of these possible combinations necessitates adaptive programming, which is crucial for ensuring patient safety and optimizing the therapeutic benefits of pacing therapy.^55^ The specifics of programming are beyond the scope of this paper, as they have been extensively detailed previously.^55^

There are several considerations concerning device configuration and programming in CRT patients with LBBP leads that require further exploration and research in the near future. These considerations are highlighted in the next paragraphs.

### Left bundle branch pacing leads for tachycardia detection in implantable cardioverter defibrillator

In patients with an indication for ICD and a LBBP-CRT strategy, the use of a DF-1 dual-chamber ICD in patients with sinus rhythm and a single-chamber ICD in patients with permanent atrial fibrillation might be considered. This particular setup requires ventricular sensing to be performed through the LBBP IS-1 lead connected to the RV port, then the defibrillation DF-1 pin is connected to the DF-1 port, and the pace-sense IS-1 component of the DF-1 lead is capped.^44,54,56,57^ An advantage of such an approach could be cost reduction and increased battery longevity. Downsides might be the fact that patients are left with capped lead and therefore a non-MRI conditional system, a higher risk for tricuspid regurgitation with two leads passing the tricuspid valve, the need for unipolar pacing (distal to the coil) configuration as a programming option which is not always possible, and the lack of specific HF algorithms present in ‘non-CRT’-ICD generators. Moreover, little is known about the detection of ventricular arrhythmias via the LBBP lead. While sensing is expected to be adequate with the LBBP lead in a healthy septal myocardium, concerns might arise regarding the safety of sensing from the LV septal region in patients with diseased myocardium.^57^ It is not unusual to observe fractionated EGMs from the LBBP lead incorporating LV and RV myocardial signals from the tip and ring in bipolar configuration.^57^ Although data so far are limited, there have been several studies showing adequate and accurate arrhythmia detection with an LBBP lead positioned in the interventricular septum with reliable and stable electrical parameters over time.^44,56,57^ Moreover, it has been reported in dogs that ATP delivered through a His lead was at least as effective and associated with less adverse outcomes (i.e. VT acceleration and VF degeneration) compared to ATP through an RV lead, possibly due to a more complete and organized capture of the myocardium during ATP delivery with a CSP lead.^58^ Studies examining new high-voltage leads intended for LBB area pacing are currently underway.

### Unipolar vs. bipolar left bundle branch pacing configuration

Left bundle branch pacing leads are typically programmed with bipolar sensing, while pacing can be unipolar or bipolar. Since LBBP targets the left bundle branch, visible tip (cathode) capture is crucial and can be achieved with unipolar programming, though this is not universally available in all ICDs. When connected to the LV port of a CRT device, an extended bipolar configuration using the LBBP tip as the cathode and the RV ring/coil as the anode can be programmed, although this may result in anodal capture at higher output.^55^

A bipolar LBBP lead tip-ring pacing configuration theoretically has the advantage of providing back-up pacing in case of LBBP lead perforation; however, anodal capture with this configuration is not always obtainable (varying between 61 and 87%)^59,60^ and necessitates higher pacing outputs.^61^ This bipolar LBBP configuration with anodal capture has been given special attention, since it has been proposed to overcome the delayed RV activation induced by LBBP. Although concerns have arisen from non-physiological RV activation, particularly during RBBB,^62^ LBBP has not demonstrated adverse effects on RV function and, in some cases, has even shown favourable remodelling in patients with baseline RV dysfunction.^63^

Studies have evaluated the electrical and haemodynamic effects of simultaneous RV and LV septal pacing via bipolar LBBP with anodal capture. In an acute human invasive study, the effects of temporary endocardial LV septal pacing alone (through *trans*-aortic approach with an EP catheter) and in combination with RV septal pacing were compared in patients undergoing CRT. No benefit was found of combined RV and LV septal pacing in this study. On the contrary, based on QRS area and LV dP/dtmax measurements, improved electrical resynchronization and haemodynamics were noticed with LVSP alone as compared to LVSP in combination with RV pacing.^64^ Further research evaluated electrical dyssynchrony with LBBP in bradycardia and CRT patients using several methods: QRS area (derived from VCG), LV activation time and SDAT (derived from ECG belt), and total electrical dyssynchrony (derived from UHF-ECG).^65^ Although LBBP with anodal capture reduced QRS duration and appeared to reduce total (interventricular) dyssynchrony, the measures of intraventricular dyssynchrony were significantly better with unipolar LBBP configuration.^65^ Studies on haemodynamic effects reported shorter QRS duration with anodal capture but no significant improvement in LV activation time or blood pressure.^61^ Finally, the CSPOT study found that bipolar LBBP resulted in anodal capture in 54% of patients but provided less LV dP/dtmax improvement than unipolar LBBP.^43^ Based on the current data, anodal capture with bipolar LBBP does not provide any proven benefit over unipolar LBBP, especially in CRT candidates.

### Fusion of intrinsic conduction with LBBP

**Figure 12:**
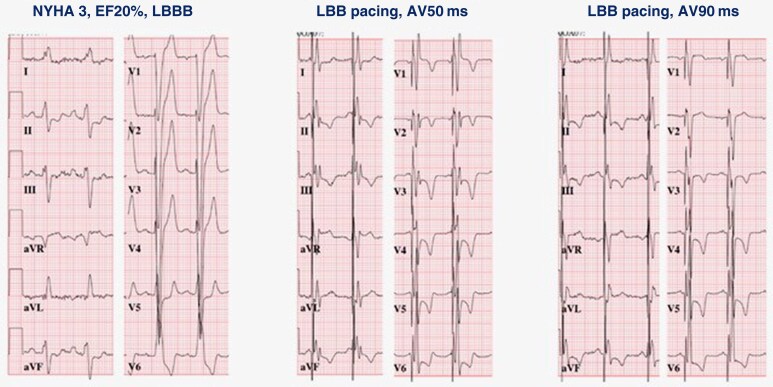
Different programmed atrio-ventricular delays during left bundle branch pacing in a patient with baseline LBBB, exhibiting fusion with intrinsic right bundle.

A solution to overcome the delayed RV activation induced by LBBP is fusion of intrinsic activation via the right bundle branch with LBBP. The AV delay can be timed to allow intrinsic right bundle conduction to fuse with LBB capture resulting in a narrow QRS (*Figure 12*). A great proportion (∼85%) of the optimal AV delays ranged from 50 to 80% of the measured atrium to LBB sensing interval in patients with LBBB.^66^ Although fusion algorithms theoretically seem to improve the delayed RV activation induced by LBBP, fusion algorithms designed specifically for CSP currently do not exist.

In CRT with LBBP only and LOT-CRT, the factors that determine the optimal timing and fusion of activation wavefronts are the conduction velocity via the His–Purkinje system, the latency of ventricular pacing at the different locations, and the intramyocardial conduction propagation. These factors require individualized programming of AV and VV delays.

## Conclusion

Conduction system pacing in patients requiring CRT can be challenging and is not always that straightforward. Adequate pre-implant evaluation and thoughtful intraprocedural decisions must be considered to tailor therapy based on the underlying substrate of the patient. Personalizing the CRT options is essential to maximize clinical benefits.

## Supplementary Material

euaf154_Supplementary_Data

## Data Availability

The data underlying this article will be shared on reasonable request to the corresponding author.
